# A new horizon of precision medicine: combination of the microbiome and extracellular vesicles

**DOI:** 10.1038/s12276-022-00748-6

**Published:** 2022-04-22

**Authors:** Jinho Yang, Tae-Seop Shin, Jong Seong Kim, Young-Koo Jee, Yoon-Keun Kim

**Affiliations:** 1 Institute of MD Healthcare Inc., Seoul, Republic of Korea; 2 Department of Internal Medicine, Dankook University College of Medicine, Cheonan, Republic of Korea

**Keywords:** Biomarkers, Biological therapy

## Abstract

Over several decades, the disease pattern of intractable disease has changed from acute infection to chronic disease accompanied by immune and metabolic dysfunction. In addition, scientific evidence has shown that humans are holobionts; of the DNA in humans, 1% is derived from the human genome, and 99% is derived from microbial genomes (the microbiome). Extracellular vesicles (EVs) are lipid bilayer-delimited nanoparticles and key messengers in cell-to-cell communication. Many publications indicate that microbial EVs are both positively and negatively involved in the pathogenesis of various intractable diseases, including inflammatory diseases, metabolic disorders, and cancers. Microbial EVs in feces, blood, and urine show significant differences in their profiles between patients with a particular disease and healthy subjects, demonstrating the potential of microbial EVs as biomarkers for disease diagnosis, especially for assessing disease risk. Furthermore, microbial EV therapy offers a variety of advantages over live biotherapeutics and human cell EV (or exosome) therapy for the treatment of intractable diseases. In summary, microbial EVs are a new tool in medicine, and microbial EV technology might provide us with innovative diagnostic and therapeutic solutions in precision medicine.

## Microbiome: Hacking microbial communication networks for clinical applications

The tiny membrane-bound vesicles containing various biomolecules that the organisms comprising our microbiome release could offer a powerful tool for precision medicine. Our bodies are home to trillions of microbes, which interact closely with our tissues to maintain a healthy physiological environment. Yoon-Keun Kim of the Institute of MD Healthcare, Seoul, South Korea, and colleagues have reviewed current research into the extracellular vesicles that these microbes use to communicate with other microbes and their human hosts. The authors note that these vesicles affect tissues throughout the body, and their activities have been linked to various disorders including asthma, Crohn’s disease and cancer. A deeper understanding of how these vesicles prevent or accelerate various conditions in different individuals could yield useful new diagnostic biomarkers and provide the foundation for interventions that are optimized for each patient.

## Introduction

The approaches to precision medicine defined by the Precision Medicine Initiative^1^ and National Research Council^2^ allow doctors and researchers to predict more accurately which treatments and preventive strategies will work in patients with a particular disease. These definitions indicate that precision medicine can guide the most effective health care decisions for a given patient and thus provide the best quality of therapy while reducing unnecessary medical interventions^3^. Precision medicine enables the individualization of patient care from diagnosis to therapy selection and monitoring using a patient’s own data in engineering technology to identify individualized treatment strategies using population-wide data^4^.

With the genomic revolution of the 21st century came the ability to rapidly sequence entire genomes in a matter of days at increasingly more affordable costs. In addition, artificial intelligence (AI) platforms have been used to optimize combination therapy in preclinical and clinical trials^4^. Thus, the prospect of deciphering an individual’s unique genome as a roadmap for precision medicine has become a tantalizing goal for scientists and physicians around the world. However, several challenges have been encountered when applying human genomic strategies in precision medicine and in translation from the bench to the clinic. To address these issues, technological improvements, including long-read sequencing methods, algorithms for indel and structural variants, graph reference approaches, and standardization of nomenclature, have been suggested^4^. The variety of next-generation methods through which precision medicine solutions have been crafted include genetic sequencing, proteomic analysis, and AI strategies. The genomic analysis ultimately remains the core foundation of most precision medicine applications.

The human genome and human microbiome projects have revealed that microbial protein-encoding genes are 360 times more abundant than human genes^5^. In 1991, Lynn and Fester^6^ defined the term “holobiont”, which is derived from Ancient Greek hólos (“whole”) and the word biont for a unit of life. In 1994, Jefferson^7^ defined the term “hologenome” when he introduced the hologenome theory of evolution at a plenary lecture at Cold Spring Harbor Laboratory. The hologenome theory of evolution reboots elements of Lamarckian evolution and recasts the individual human as a community or a holobiont that is the host plus all of its symbiotic microbiota species^8,9^. Variation in the hologenome may encode phenotypic plasticity of the holobiont and can be subject to evolutionary changes caused by selection and drift if portions of the hologenome are transmitted between generations with reasonable fidelity. One of the important outcomes of recasting the individual as a holobiont subject to evolutionary forces is that genetic variation in the hologenome can be brought about by changes in the host genome and microbiome, including new acquisitions of microbes, horizontal gene transfers, and changes in microbial abundance within hosts. Thus, humans might be holobionts, with 99% of their DNA attributed to microbial genomes (the microbiome) and 1% of their DNA attributed to the human genome, according to the hologenome theory of evolution.

Extracellular vesicles (EVs) are lipid bilayer-delimited particles that are naturally released from almost all types of cells and cannot replicate. The vast majority of EVs are smaller than 200 nm. EVs, according to the biogenesis route, are divided into exosomes, ectosomes, and apoptotic bodies^10^. EVs carry cargoes of proteins, nucleic acids, lipids, metabolites, and even organelles from parent cells. Most cells that have been studied to date are thought to release EVs, including archaeal, bacterial, fungal, plant, and human cells^11^. Microbial EVs (MEVs) appear to be key messengers in host cell−microbiota communication. Recent experimental evidence shows that MEVs are key messengers in the communication between host cells and the microbiota. In this review, we will address MEVs as a new factor in precision medicine, focusing on 1) MEV biogenesis and functions, 2) the importance of MEVs in disease pathogenesis, and 3) the application of MEVs in the development of diagnostic and therapeutic solutions for precision medicine.

## Microbial EV biogenesis

**Fig. 1:**
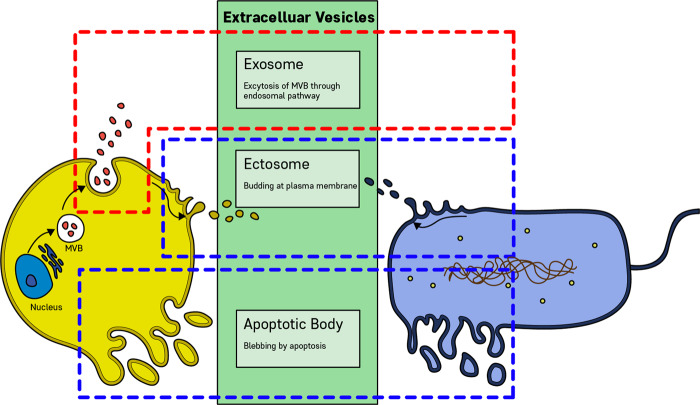
Biogenesis of extracellular vesicles (EVs) derived from eukaryotic cells and prokaryotic cells. Extracellular vesicles can be classified into three main classes: Exosome, exosomes are formed within the endosomal network and released upon fusion of multivesicular bodies (MVBs) with the plasma membrane. Ectosomes, ectosomes are produced by outward budding and fission of the plasma membrane. Apoptotic bodies, apoptotic bodies are released as blebs of cells by apoptosis. Eukaryotic cells can release exosomes, ectosomes, and apoptotic bodies, whereas prokaryotic cells are able to secrete ectosomes and apoptotic bodies.

**Fig. 2:**
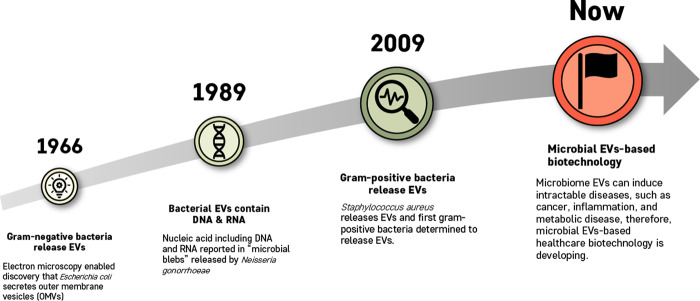
Key milestones of microbial EV research. The main findings of such studies are that gram-negative bacteria release EVs, bacterial EVs contain DNA and RNA, and gram-positive bacteria release EVs. Currently, microbial EV-based biotechnology is developing dramatically.

The biogenesis of EVs is a very tightly regulated process governed by multiple signaling molecules and begins with receptor activation that is unique for each cell type^12^. Eukaryotic EV biogenesis is well characterized, whereas MEV biogenesis has only recently been elucidated^13^. According to the method of biogenesis, eukaryotic cell-derived EVs are classified as exosomes, ectosomes (or shedding vesicles), and apoptotic bodies^14,15^, while prokaryotic cell-derived EVs are classified as ectosomes and apoptotic bodies^16^ (Fig. 1). Both gram-negative and gram-positive bacteria produce ectosomes, known as outer membrane (OM) vesicles (OMVs) and membrane vesicles (MVs), respectively^16^. The key milestones of bacterial EVs are as follows (Fig. 2): (1) gram-negative bacteria-derived OMVs were first discovered in 1966^17^, (2) the fact that bacterial EVs contain nucleic acids was reported in 1989^18^, (3) gram-positive bacteria-derived EVs were first included in a publication in 2009^19^, and (4) since 2010, much evidence has shown that both gram-negative and gram-positive bacteria release EVs that are positively or negatively involved in disease pathogenesis.

When OMVs are released from gram-negative bacteria during their proliferation, the OM bulges outwardly, and the connection between the OM and peptidoglycans is broken. The next steps are an accumulation of peptidoglycan fragments and misfolded proteins in the periplasmic space and enrichment of species-specific membrane curvature-inducing molecules^16,20^. However, this mechanism cannot sufficiently explain OMV biogenesis. Thus, new mechanisms for OMV formation have been reported. Several studies have shown that overexpressed and misfolded periplasmic proteins can cause budding, and specific proteins have been found to be enriched in or excluded from OMVs, suggesting a more sophisticated biogenesis pathway. The accumulation of periplasmic proteins, as well as the accumulation of curvature-inducing OM proteins, induces additional budding events^21,22^. Another study showed that the VacJ/Yrb ATP-binding cassette transport system was involved in OMV formation. In that report, a decrease in or deletion of the VacJ or Yrb genes was found to result in phospholipid accumulation in the outer leaflet of the OM. This asymmetric expansion of the outer leaflet then caused an outward bulging of the OM. Then, further enrichment of the positive and negative curvature-inducing phospholipids in both leaflets supported the budding of the OM. Ultimately, the released OMV was enriched in phospholipids incorporated into the outer leaflet of the vesicle membrane^20,23^.

Gram-positive bacteria lack an OM and have a much thicker peptidoglycan cell wall. Thus, until 2009, it was assumed that gram-positive bacteria could not release EVs. However, recent experimental evidence has shown that gram-positive bacteria release MVs^19,24,25^. The biogenesis mechanisms of MVs during the proliferation of gram-positive bacteria have not been as clearly elucidated compared to those of gram-negative bacterial ectosome biogenesis. One hypothesis is that MVs may be forced through the cell wall by turgor pressure after their release from the plasma membrane^26^. Another hypothesis is that cell walls loosened by enzymes may enable the release of MVs. For example, *Staphylococcus aureus (S. aureus)*-derived MVs carry peptidoglycan-degrading enzymes, which can modify thick gram-positive peptidoglycan cell walls^19^. Thus, membrane fluidity and cell wall integrity, affected by peptidoglycan cross-linking, might be critical for the release of MVs^25^. The third hypothesis is that EVs may pass through channels such as protein channels or structural cables from the intracellular to the extracellular environment^26^.

Regarding the biogenesis of apoptotic bodies, much evidence has revealed that bacterial cell apoptosis is induced in response to environmental stress, biofilm formation, and genetic transformation for the benefit of the entire colony rather than the individual cell^27^. Once the programmed cell death signaling pathways are activated, DNA damage and fragmentation are induced, and subsequently, cell membrane blebs, are induced, resulting in cell fragmentation and the release of apoptotic bodies^15^.

### Microbial EV components and physiological functions

Nanometer-sized MEV particles contain a variety of functional components, including lipids, proteins, nucleic acids, and metabolites, within a spherical phospholipid bilayer and play a key role in cell-to-cell communication^10,16,21,26^.

#### Membrane components: lipids, carbohydrates, and proteins

Lipids in MEVs can play roles as drug delivery vehicles. The bilayer lipids of OMVs mainly contain lipopolysaccharide (LPS) and OM lipids^28^. Liposomal vesicles can be incorporated into cultured mammalian cells with no cytotoxic effects. The variety of glycolipids and glycoproteins in liposomal vesicles suggests the potential to modify the cellular composition and introduce biologically active molecules into cells^16,29^. In addition, the asymmetric distribution of EV lipids suggests that EVs could be synthesized in lipid domains, which could potentiate a lipoprotein-dependent effect on cargo loading^25^. For example, lipid domains, such as functional membrane microdomains, were found to exist in the membranes of *Bacillus subtilis*^30,31^. Therefore, EVs have been considered advantageous as pharmacological vehicles.

Lipids, carbohydrates, and proteins in the MEV membrane are crucial for specific targeting. In particular, the physical state of the lipids in EVs is important for determining the pathways of EVs^16^. The EV lipid bilayer is adapted to different target environment conditions, such as pH, for optimal function^32^. The carbohydrates in the cell membrane include receptors for glycoproteins, suggesting that the tissue distribution and cellular uptake of phospholipid EVs could be controlled by carbohydrate determinants on the EV surface^33,34^. Modification of the EV surfaces with specific synthetic glycolipids significantly affected the tissue distribution and stability of the EVs in mice^35,36^. Furthermore, the recognition systems of cell membrane carbohydrates include receptors for galactose-terminated glycoproteins in hepatocytes, 6-phosphomannosyl residue-containing glycoproteins in fibroblasts, and mannose-terminated glycoproteins in macrophages^16,37–39^. These membrane glycoprotein receptors are essential for specific targeting. Generally, membrane proteins are essential components for EV functions, such as immunity, targeting, and pathogenicity, and EV proteins can play significant roles in regulatory processes, cellular responses, host−microbe interactions, and targeting^32^. OM proteins have been shown to participate in flagellum assembly, pore formation, transport of specific substrates, OM stabilization, antibiotic resistance, proteolysis, and host bacteria interaction^28^.

#### Luminal components: proteins, nucleic acids, and metabolites

Proteomics studies have shown that there are a number of proteins in the membrane or luminal space of MEVs, which demonstrate complex protein organization^19,25,40,41^. Most EV membrane proteins originate from the cytoplasmic membrane, while EV luminal proteins are cytoplasmic proteins packaged during vesiculogenesis^25^. For example, *S. aureus* EVs (SaEVs) were found to have more than 200 proteins associated with EVs, and 160 of these proteins were cytoplasmic proteins^42^. Furthermore, α-hemolysin is localized in the lumen of SaEVs^43^. α-Hemolysin in EVs induces necrotic cell death via toxin entry into the cytoplasm of keratinocytes, whereas soluble α-hemolysin induces keratinocyte death. Thus, α-hemolysin in the EV lumen enhances keratinocyte death and the evasion of host immune defenses, illustrating the clinical significance of EVs in luminal protein delivery^43^.

Previous studies have shown that MEVs carry DNA, rRNA, tRNA, mRNA, and sRNA^16,25^. The majority of RNAs in EVs are sRNAs and are noncoding^16,44^. In addition, a large proportion of sRNAs in EVs are from uncharacterized intergenic regions and may also modulate gene expression in target cells^44,45^. However, there is much less evidence regarding the properties and functions of the nucleic acids in MEVs^44,46^. Further studies using next-generation sequencing (NGS) and transcriptome sequencing are needed to ascertain the content and function of MEV nucleic acids^25^. Previous studies suggest that the nucleic acids in MEVs could interfere with the host transcriptome and inhibit protein synthesis^25^.

The nucleic acids in EVs mediate horizontal gene transfer and immunomodulatory effects^16^. To affect gene expression in target cells directly, RNAs in EVs enter the cytoplasm. Once in the cytoplasm, microbial RNAs might interact with or be modified by target cell factors. Complementarity between sRNAs in EVs and RNAs in target cells might induce the attenuation or enhancement of gene expression via transcription, translation, or mRNA processing. The mechanisms involved likely vary greatly depending on the specific RNAs in EVs and the presence of specific target cell factors. mRNAs in EVs might be translated to produce microbial proteins in target cells^44^. Microbial nucleic acids are sensed by either endosomal or cytoplasmic receptors in host cells^47^. Several MEVs trigger pattern recognition receptor signaling to promote protective immunity, a property that has been harnessed for vaccine development^44^.

Recently, it has been reported that EVs are metabolically active^16^. Although emerging evidence suggests that EVs can act as metabolic regulators, the involvement of EVs in metabolic activity and the existence and function of metabolites in MEVs have not been completely characterized^16^. Thus, it is necessary to further investigate the metabolites in MEVs to demonstrate their potential metabolic functions.

### Microbial EVs in pathogenesis

Previous studies elucidated the role of MEVs in disease development and the pathogenesis of diseases, including skin, lung, and gut diseases, metabolic diseases, and cancers (Table 1).

**Table 1:** Role of microbial EVs in pathogenesis.

Organ	Disease	EV species of origin	Pathogenic effects	Ref.
Skin	Atopic dermatitis	*Staphylococcus aureus (S. aureus)*	The application of *S. aureus* EVs induced activation of dermal fibroblasts, increasing the production of pro-inflammatory mediators such as IL-6, thymic stromal lymphopoietin, macrophage inflammatory protein-1a, and eotaxin. In addition, the application of *S. aureus* EVs caused epidermal thickening with infiltration of the dermis by mast cells and eosinophils, which are associated with the enhanced cutaneous production of IL-4, IL-5, IFN-c, and IL-17.	Hong et al.^121^
			α-Hemolysin in *S. aureus* EVs was cytotoxic to HaCaT keratinocytes and induced necrosis. In addition, α-hemolysin in *S. aureus* EVs induced skin barrier disruption and epidermal hyperplasia and caused dermal inflammation.	Hong et al.^48^
	Acne vulgaris	*Propionibacterium acnes (P. acnes)*	*P. acnes* EVs significantly increased the secretion of cytokines, such as IL-8, GM-CSF, CXCL-1, and CXCL-5, promoting the infiltration of neutrophils. In addition, *P. acnes* EVs induce dysregulated epidermal differentiation of human keratinocytes via TLR2-mediated signaling pathways. *P. acnes* EVs may play pivotal roles in the early stages of the development of acne vulgaris by initiating inflammatory cytokine cascades in keratinocytes.	Choi et al.^50^
Lung	Asthma or chronic obstructive pulmonary disease (COPD)	*Staphylococcus aureus (S. aureus)*	After stimulation with *S. aureus* EVs, alveolar macrophages induced both TNF-a and IL-6 production, and airway epithelial cells induced only IL-6 production. Repeated airway exposure to *S. aureus* EVs induced both Th1 and Th17 cell responses and neutrophilic pulmonary inflammation, mainly via a TLR2-dependent mechanism. In terms of adjuvant effects, airway sensitization with *S. aureus* EVs and OVA resulted in neutrophilic pulmonary inflammation after OVA challenge alone. This phenotype was partly reversed by the absence of IFN-γ or IL-17. Neutrophilic inflammation led to airway hyperreactivity and fibrosis that contributed to asthma development, and the combination of increased elastase production and fibrosis caused COPD.	Kim et al.^55^
	Pulmonary inflammation	*Pseudomonas aeruginosa (P. aeruginosa)*	*P. aeruginosa* EVs increased the concentrations of several chemokines and cytokines in the lungs and alveolar macrophages. The inflammatory responses to *P. aeruginosa* EVs were partly regulated by the TLR2 and TLR4 pathways. *P. aeruginosa* EVs cause pulmonary inflammation, which was only partly controlled by TLR2 and TLR4. Furthermore, *P. aeruginosa* EVs caused dose- and time-dependent pulmonary cellular inflammation.	Park et al.^57^
	Emphysema	*Escherichia coli (E. coli)*	Airway exposure to *E. coli* EVs increased the production of pro-inflammatory cytokines, such as TNF-a and IL-6. In addition, the repeated inhalation of *E. coli* EVs induced neutrophilic inflammation and emphysema, and elastase activity was enhanced. Emphysema and elastase activity were reversed by the absence of the IFN-γ or IL-17A genes. In addition, during the early period, lung inflammation was dependent on IL-17A and TNF-a. Moreover, the production of IFN-γ was eliminated by the absence of IL-17A, whereas IL-17A production was not abolished by IFN-γ absence. In summary, *E. coli* EVs induced IL-17A-dependent neutrophilic inflammation and thereby emphysema via upregulation of elastase activity.	Kim et al.^54^
	Lung fibrosis	*Bacteroides ovatus (B. ovatus), Bacteroides stercoris* *(B. stercoris), Prevotella melaninogenica (P. melaninogenica)*	The EVs of three bacterial species (*B. ovatus, B. stercoris, and P. melaninogenica*) induced IL-17B expression and consequently promoted Th17 cell development via TLR-Myd88 adaptor signaling. The expanded lung microbiota induced IL-17B expression and consequently Th17 cell expansion to promote lung fibrosis.	Yang et al.^58^
	Chronic inflammatory airway diseases	*Staphylococcus aureus (S. aureus), Escherichia coli (E. coli)*	*E. coli* EVs and *S. aureus* EVs induced MUC5AC expression via the ERK1/2 and p38 MAPK signaling pathways in human airway epithelial cells. *E. coli* EVs and *S. aureus* EVs significantly activated phosphorylation of ERK1/2 MAPK and p38 MAPK. An ERK1/2 MAPK inhibitor, a p38 MAPK inhibitor, an ERK1/2 MAPK siRNA, and a p38 MAPK siRNA significantly blocked the MUC5AC mRNA expression induced by *E. coli* EVs and *S. aureus* EVs.	Bae et al.^56^
Gastrointestinal	Gastric disease	*Helicobacter pylori (H. pylori)*	*H. pylori* and *H. pylori* EVs induced the expression of IL-8 mRNA and protein. IL-8 expression was induced to different levels in response to *H. pylori* EVs from hosts with different gastric diseases. Exposure to *H. pylori* EVs increased the phosphorylation and reduced the degradation of inhibitor of NF-κB alpha. *H. pylori* EVs may aid the development of various gastric diseases by inducing IL-8 and NF-κB expression.	Choi et al.^69^
	Inflammatory bowel disease	*Vibrio cholera (V. cholerae)*	*V. cholerae* EVs activated NOD1 and NOD2, resulting in NF-κB signaling and a pro-inflammatory immune response. NOD1 and NOD2 activation by EVs is of particular interest for inflammatory diseases such as Crohn’s disease, in which targeting NOD signaling may be an effective therapeutic strategy.	Bielig et al.^66^
		*Escherichia coli (E. coli)*	*E. coli* EVs activated NOD1 signaling pathways in intestinal epithelial cells. NOD1 silencing and RIP2 inhibition significantly abolished EV-mediated activation of NF-κB and subsequent IL-6 and IL-8 expression. In addition, endocytosed EVs colocalized with NOD1, triggered the formation of NOD1 aggregates, and promoted NOD1 association with early endosomes.	Canas et al.^67^
Metabolic	Type 2 diabetes (T2D)	*Pseudomonas panacis (P. panacis)*	*P. panacis* EVs blocked the insulin signaling pathway in both skeletal muscle and adipose tissue, thereby promoting glucose intolerance in skeletal muscle. Moreover, *P. panacis* EVs induced typical diabetic phenotype characteristics, such as glucose intolerance after glucose administration or systemic insulin injection. *P. panacis* EVs induced T2D via the induction of insulin resistance in insulin-responsive organs.	Choi et al.^71^
	Diabetes mellitus	*Porphyromonas gingivalis (P. gingivalis)*	*P. gingivalis* EVs equipped with gingipain proteases were translocated to the liver. In addition, the hepatic glycogen synthesis in response to insulin was decreased, and thus, high blood glucose levels were maintained. *P. gingivalis* EVs also attenuated insulin-induced Akt/GSK-3β signaling in a gingipain-dependent fashion in hepatic HepG2 cells. The delivery of gingipains mediated by *P. gingivalis* EV elicited changes in glucose metabolism in the liver and contributed to the progression of diabetes mellitus.	Seyama et al.^72^
Cancer	Stomach cancer	*Helicobacter pylori (H. pylori)*	*H. pylori* EVs contained CagA and VacA. They induced the production of TNF-α, IL-6 and IL-1β by macrophages and IL-8 by gastric epithelial cells. Additionally, *H. pylori* EVs induced the expression of IFN-γ, IL-17, and *H. pylori* EV-specific IgG1. *H. pylori* EVs infiltrated and remained in the stomach for an extended time. *H. pylori* EVs, which are abundant in the gastric juices of gastric cancer patients, can induce inflammation and possibly cancer in the stomach, mainly via the production of inflammatory mediators from gastric epithelial cells after selective uptake by the cells.	Choi et al.^77^
	Lung cancer	*Staphylococcus aureus (S. aureus) Escherichia coli (E. coli)*	*S. aureus* and *E. coli* EVs triggered the Th17 response. Generally, *S. aureus* and *E. coli* EVs induced IL-17 production through polarized Th17 cells and thus caused neutrophilic inflammation. The neutrophilic inflammatory response induces epithelial cell dysplasia and MMP expression, which can lead to lung cancer.	Yang et al.^53^
Central nervous system	Alzheimer’s disease	*Paenalcaligenes hominis (P. hominis)*	Oral administration of *P. hominis* EVs caused cognitive impairment in mice. *P. hominis* EV treatment increased NF-κB+/Iba1+, LPS+/Iba1+, and IL-1R+ cell counts in the hippocampus. *P. hominis* EV treatment reduced BDNF expression in the hippocampus while increasing IL-1β expression in the blood. Vagotomy significantly reduced the occurrence of cognitive impairment caused by *P. hominis* EV gavage. Vagotomy inhibited EV-induced changes in NF-κB+/Iba1+, LPS+/Iba1+, and IL-1R+ cell populations in the hippocampus. The occurrence of cognitive impairment induced by *P. hominis* EVs was significant. Fluorescein isothiocyanate (FITC)-conjugated EVs were detected in the pyramidal region of the hippocampus. However, vagotomy significantly reduced the FITC-conjugated EV-containing CD11c+ cell population. Furthermore, oral gavage of *P. hominis* EVs increased bacterial 16S rDNA levels in the hippocampus. The translocation of *P. hominis* EVs through the vagus nerve resulted in cognitive impairment induced by brain inflammation.	Lee et al.^81^
		*Aggregatibacter actinomycetemcomitans (A. actinomycetemcomitans)*	*A. actinomycetemcomitans* EVs administered via intracardiac injection into mice were successfully delivered to the brain after crossing the blood–brain barrier, and the extracellular RNA cargos increased the expression of TNF-α through the TLR-8 and NF-κB signaling pathways in the mice. Thus, host gene regulation by microRNAs originating from *A. actinomycetemcomitans* EVs is a novel mechanism for host gene regulation, and transfer of *A. actinomycetemcomitans* EV extracellular RNAs to the brain may cause neuroinflammatory diseases like Alzheimer’s disease.	Han et al.^79^

*GM-CSF* granulocyte−macrophage colony-stimulating factor, *ERK* extracellular signal-regulated kinase, *MAPK* mitogen-activated protein kinase, *NOD* nucleotide oligomerization domain, *NLR* NOD-like receptor, *MMP* matrix metalloproteinase.

#### Skin diseases

Atopic dermatitis (AD) is a chronic inflammatory disease of the skin and is defined as eczematous lesions with pruritus and xerosis. AD skin lesions show distinct features, such as abnormal skin barrier function with epidermal hyperplasia and *S. aureus* colonization. Abnormal skin barrier function induced by the death of keratinocytes is one of the major causative factors of AD^43^. Skin inflammation has been associated with SaEVs^43,48^. SaEVs increase IL-6, IL-17, serum IgG1, IgE, thymic stromal lymphopoietin, macrophage inflammatory protein-1α, and eotaxin levels and recruit mast cells and eosinophils in the skin^48^. A previous study on the metagenomics of MEVs showed that *Staphylococcus* accounted for the highest proportion of bacterial genera in AD patients, and the proportion of *Staphylococcus* significantly decreased after treatment^49^.

If the skin barrier is disrupted, pathogens can penetrate the human body. Therefore, skin barrier disruption is a major cause of AD exacerbation^43^. α-Hemolysin can induce keratinocyte cell death, consequently enhancing skin penetration of high-molecular-weight allergens. SaEV-associated α-hemolysin was found to induce keratinocyte necrosis and induce epidermal thickening and eosinophilic inflammation in the dermis. SaEVs were internalized into the cytoplasm of keratinocytes, and EVs efficiently delivered α-hemolysin to the cytoplasm. These findings suggest that EV-associated toxins are key molecules in disease pathogenesis^43^. Therefore, SaEVs are useful in the development of in vivo models of AD^49^. *Propionibacterium acnes (P. acnes)* EVs induce dysregulated epidermal differentiation in human keratinocytes via TLR2-mediated signaling pathways. *P. acnes* EVs may play pivotal roles in the early stages of the development of acne vulgaris^50^.

#### Lung diseases

The main causative factor of chronic inflammatory diseases in the lung has been suggested to be exposure to chemical substances such as cigarette smoke and air pollution^51,52^ because of the inherent pharmacological or toxic effects of such chemical substances. However, the etiology of a large proportion of chronic inflammatory diseases is not related to these factors. Biological factors, including allergens, viruses, and bacterial factors, can cause severe inflammatory reactions^52^.

Airway exposure to MEVs triggers two main pathophysiological mechanisms, Th17, and Th1 responses, based on whether the parent bacterial cell is extracellular or intracellular, respectively. Specifically, extracellular bacteria-derived EVs generally cause neutrophilic inflammation through IL-17 release by polarized Th17 cells, whereas intracellular MEVs induce mononuclear inflammation through IFN-γ produced by polarized Th1 cells^53^. The pathogenic MEV-induced neutrophilic inflammatory response leads to airway hyperreactivity and fibrosis that contribute to asthma development. The combination of increased elastase production and fibrosis induced by neutrophilic inflammation can cause emphysema, which is a key pathology of chronic obstructive pulmonary disease (COPD). Furthermore, intracellular MEVs lead to Th1 polarization and subsequent IFN-γ-induced mononuclear inflammation, leading to increased elastase production in the alveoli and causing pulmonary tuberculosis-associated emphysema^53^.

In several in vivo tests, MEVs caused severe airway inflammation, which resulted in severe asthma and emphysema^52^. Previous studies have shown that repeated exposure to *Escherichia coli (E. coli)* EVs (EcEVs) in the airways led to IL-17-dependent neutrophilic inflammation and emphysema in conjunction with elastase upregulation^54^. Repeated airway exposure to SaEVs induced both Th1 and Th17 immune responses with IFN-γ and IL-17, respectively, and neutrophilic pulmonary inflammation was increased primarily through TLR2 engagement^55^. EcEVs and SaEVs induced MUC5AC expression in human airway epithelial cells. MUC5AC, a major mucin, is overexpressed in chronic inflammatory airway diseases and causes airway obstruction, increases susceptibility to infection, and decreases pulmonary function^56^. In addition, *Pseudomonas aeruginosa* EVs increase pulmonary inflammation through activation of the TLR2 and TLR4 pathways through dose-dependent increases in chemokines (CXCL1 and CCL2) and cytokines (IL-1β, TNF-α, IL-6, and IFN-γ), as well as increases in neutrophils and macrophages^57^. In addition, EVs from *Bacteroides ovatus, Bacteroides stercoris*, and *Prevotella melaninogenica* induce IL-17 expression and consequently promote Th17 cell development associated with lung fibrosis^58^.

Indoor dust containing MEVs induces both Th1 and Th17 responses associated with neutrophilic pulmonary inflammation^59,60^ and over 50% of the patients with childhood asthma were found to be sensitized to MEVs in indoor dust, suggesting that MEVs in indoor dust could be an important causative factor in the pathogenesis of childhood asthma^59^. Moreover, the anti-indoor dust EV IgG antibody was found to be significantly associated with the risk of asthma, COPD, and lung cancer (LC)^61^. These results suggest the potential of MEVs as a major risk factor in the development of inflammatory diseases and cancer in the lung.

#### Gastrointestinal disease

Inflammatory bowel disease (IBD) is characterized by chronic inflammation of the gastrointestinal tract. Although the exact etiology of IBD is under investigation, disruption of the normal intestinal flora and normal intestinal function have been linked to the development of IBD. Alteration of the commensal gut microbial ecosystem, which interacts with gut epithelial cells and plays significant roles in the maintenance of the mucosal barrier, induces dysfunctional immunomodulation and metabolic activity in the gut^62^. Such disruptions lead to reductions in mucus barrier functionality, including decreased tight junctions in the intestinal epithelial lining. Since the integrity of the mucosal barrier is significantly reduced, inflammatory agents such as microbial components in the intestinal lumen can induce mucosal inflammation associated with IBD^63,64^.

The NOD-like receptor (NLR) family, which is a family of cytoplasmic pattern recognition receptors, initiates inflammation by activating signaling pathways, including NF-κB signaling, followed by induction of caspase-1-mediated cleavage of IL-1β/IL-18 and production of gasdermin, which causes pyroptotic cell death via the formation of pores in the plasma membrane. NLRs or NLR-associated proteins have been found to be genetically associated with susceptibility to IBD^65^. *Vibrio cholerae* EVs and EcEVs activate NOD1 signaling^66,67^, resulting in NF-κB signaling and a pro-inflammatory immune response. NOD1 activation by MEVs can lead to IBDs, such as Crohn’s disease^68^.

*Helicobacter pylori* (*H. pylori*) EVs (HpEVs) may play a significant role in the development of various gastric diseases through IL-8 production and NF-κB activation. HpEVs may play a crucial role in the pathogenesis of gastric disease associated with *H. pylori*^69^.

#### Metabolic diseases

Commensal bacteria in the gastrointestinal tract play an important role in nutrient absorption and the fermentation of dietary fibers. In addition, the gut microbiota provides a critical function in the production of short-chain fatty acids (SCFAs) for energy sources and epigenetic regulation via histone deacetylase inhibition^70^. Gut microbiota dysbiosis resulting in irregular SCFA production can lead to dysregulated host metabolic functionality. Moreover, MEVs can participate in the development of metabolic diseases. *Pseudomonas panacis* EVs promoted by a high-sucrose/fat diet can induce type 2 diabetes (T2D) via the induction of insulin resistance in insulin-responsive organs. *Pseudomonas panacis* EVs blocked the insulin signaling pathway in both skeletal muscle and adipose tissue, thereby promoting glucose intolerance in skeletal muscle, while these EVs induced typical diabetic phenotype characteristics, such as glucose intolerance after glucose administration or systemic insulin injection^71^.

In one study, *Porphyromonas gingivalis* EVs equipped with proteases gingipains were translocated to the liver. Subsequently, hepatic glycogen synthesis in response to insulin was decreased, and high blood glucose levels were maintained. *Porphyromonas gingivalis* EVs also attenuated insulin-induced Akt/GSK-3β signaling in a gingipain-dependent fashion in hepatic HepG2 cells. The delivery of gingipains mediated by *Porphyromonas gingivalis* EVs elicited changes in glucose metabolism in the liver and contributed to the progression of T2D^72^. These results suggest a close relationship between MEVs and metabolic disease.

#### Cancers

MEVs are known to be associated with tumors, and cancer patients show different MEVs than healthy people. Previous studies have elucidated that Th17 cells play an important role in pathogen clearance during host defense reactions but induce tissue inflammation in the pathogenesis of autoimmune disease^73^. Recent studies reported that Th17 cells played an important role in LC pathogenesis^74,75^, and the immune effector T cells in small-cell LC patients included a large proportion of Th17 cells^76^. Airway exposure to SaEVs and EcEVs triggers the Th17 response. Generally, OMVs induce IL-17 production through polarized Th17 cells and thus cause neutrophilic inflammation. The neutrophilic inflammatory response induces epithelial cell dysplasia and the induction of matrix metalloproteinase expression, possibly leading to LC. COPD also increases the risk of LC^53^.

The abundances of *H. pylori* and HpEVs in gastric cancer patients were significantly higher than those in healthy people. HpEVs contain CagA and VacA proteins that can induce the production of TNF-α, IL-6, IL-1β, and IL-8. In addition, HpEVs induce the expression of IFN-γ, IL-17, and EV-specific IgGs in mice. In summary, HpEVs might be involved in the pathogenesis of gastric cancer, mainly via the induction of the production of inflammatory mediators by gastric epithelial cells after selective uptake by the cells^77^.

#### Central nervous system (CNS) diseases

MEVs can penetrate the blood–brain barrier (BBB) through three possible mechanisms: (1) receptor-mediated transcytosis, (2) paracellular passage in disease states, or (3) via EVs in infected immune cells. Once MEVs are inside brain tissues after penetrating the BBB, the components and cargo in EVs act as ligands of innate immune receptors, such as TLRs, and the NALP3 inflammasome, and activate the inflammatory immune response^78^.

A previous study showed that *Aggregatibacter actinomycetemcomitans* EVs can cross the mouse BBB and are present in the brain 24 h after intracardiac injection, inducing TNF-α production via extracellular RNAs through the TLR-8 and NF-κB signaling pathways^79^. In the mouse brain, increased TNF-α may cause neuroinflammatory diseases, such as Alzheimer’s disease^79^. Similarly, *Aggregatibacter actinomycetemcomitans* EVs can successfully deliver extracellular RNAs to brain monocytes/microglial cells to cause neuroinflammation associated with the upregulation of IL-6 through NF-κB activation^80^. Oral administration of *Paenalcaligenes hominis* EVs increased bacterial 16 S rDNA levels in the hippocampus, causing cognitive impairment in mice, whereas oral administration of LPS caused more severe cognitive impairment. *Paenalcaligenes hominis* EV treatment increased NF-κB+/Iba1+, LPS+/Iba1+, and IL-1R+ cell counts and reduced BDNF expression in the hippocampus while increasing IL-1β expression in the blood^81^. These findings provide evidence that some MEVs are important in the pathogenesis of neuroinflammatory diseases.

Recently, we performed microbiome analysis of EVs in blood and brain tissue samples from brain tumor (BT) patients and healthy controls (HCs)^82^. Blood EV microbiome analysis at the phylum level showed that the abundance of Firmicutes was significantly higher in BT patients than in HCs, whereas those of Proteobacteria and Actinobacteria were significantly lower. In addition, at the genus level, the proportions of EVs from *Ruminococcaceae UCG-014, Lachnospiraceae NK4A136, Lactobacillus, Ruminococcaceae UCG-013, Ruminiclostridium 6*, and *Peptoclostridium* were significantly higher but those from *Escherichia-Shigella, Blautia, Bifidobacterium, Streptococcus*, and *Sphingomonas* were significantly lower in BT patients than in HCs. Moreover, brain tissue microbiome analysis at the genus level showed that the abundance of EVs from *Bacteroides* and *Erysipelatoclostridium* was significantly higher but those from *Chloroplast(c), Prevotella 9*, and *Saccharibacteria(p)* were significantly lower in BT patients than in HCs^82^. These findings suggest that MEVs might be closely linked to the pathogenesis of CNS diseases, although further research is needed.

### Microbial EVs as biomarkers

**Fig. 3:**
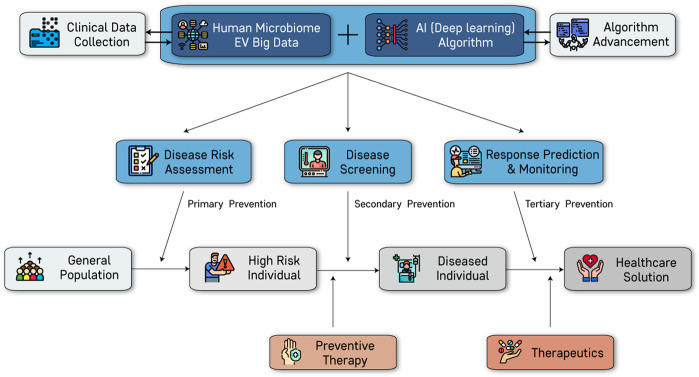
Application of microbial EVs for precision medicine. Precision medicine goals, including risk assessment, screening, prediction, and monitoring, are based on analysis using an artificial intelligence (AI) algorithm through human microbial EV big data and clinical data. High-risk individuals and diseased individuals can be identified by risk assessment, and preventive therapy can reduce disease risk. Diseased individuals identified by screening and diagnosis are suggested therapeutics based on the results of response prediction and monitoring. These processes can realize microbial EV-based health care solutions.

Liquid biopsy, also known as fluid biopsy or fluid phase biopsy, is the sampling and analysis of nonsolid biological tissue. MEVs are a newly emerging biomarker that can be attested for by liquid biopsy. Moreover, AI-based analysis of clinical and MEV data enables the development of a more accurate prediction of disease status (Fig. 3).

Commensal microbes secrete MEVs for cell-to-cell communication. MEVs penetrate the mucosal barrier, circulate throughout the body, and then are excreted via feces, urine, and exhaled air in their intact forms, unlike live microbes, which are restricted to the mucosal lumen or skin surface^71^. Certain MEVs act as etiological agents of various diseases, while some MEVs have a protective role in disease pathogenesis. Thus, MEVs circulating in our body provide us with good information for health and disease status.

An organism’s evolutionary history is documented in its genome. rDNA encoding rRNA changes slowly and is useful for investigating relationships between taxa that diverged hundreds of millions of years ago. In contrast, mitochondrial DNA evolves rapidly and is more useful for exploring more recent evolutionary events. MEVs harbor 16S rDNA, which provides taxonomy information^83^. However, there are some issues in comparing published microbiome data. First, the regions targeted during the investigation of the microbiota are different for the 16S rDNA and NGS platforms. The diversity and abundance of the bacterial mock community are different between the V1-V3 region and V3-V4 region of the 16S rRNA or MiSeq platform (Illumina, USA) and the Roche 454 platform (Roche, Switzerland)^84^. Second, microbiome information differs among the databases used for taxonomic assignments. There are several public databases, such as SILVA, RDP, Greengenes, EzTaxon, and NCBI, and these databases have different taxonomic classifications^85^. Third, these public databases are updated continuously, and unassigned taxa have the potential to be assigned in the future.

Disease diagnosis and prediction goals, such as risk assessment, early diagnosis, treatment response prediction, and disease monitoring, are important for the reduction of mortality and the increase of quality of life^86,87^. MEVs are novel biomarkers for in vitro diagnostics. Several 16S rDNA amplicon studies using serum, urine, feces, and skin-fluid samples have shown significant differences in MEV composition between healthy and patient groups (Table 2). The clinical performance of the diagnostic models as evaluated by area under the curve (AUC) analysis using external validation set was 0.97, 1.00, 0.95, 0.93, 0.87, 0.82, 1.00, and 1.00 for asthma^88^, AD^89^, colorectal cancer^90^, BT^82^, hepatocellular carcinoma^91^, gastric cancer^92^, pancreatic cancer^93^, and biliary tract cancer^94^, respectively. Furthermore, diagnostic models were developed to predict the prognosis of hepatic diseases^91^. MEV-based models, in combination with microbiome and metabolome data, provided the best performance; the performance of colorectal cancer models using both metagenomic and metabolomic data was better than that of models using microbiome or metabolome data alone^90^. Moreover, EV microbiome data provided better performance when combined with tumor markers; the diagnostic performance in ovarian cancer was better using both MEV data and the tumor marker CA-125 than either biomarker alone^95^.

**Table 2:** Diagnostic models using microbiome data derived from EVs.

Patient group	Sample type	Genera		Diagnostics		Ref.
		Enriched in case	Enriched in control	Method	Performance	
Colorectal cancer	Feces	*Faecalibacterium, Eubacterium, Ruminococcus, Bifidobacterium*	*Escherichia-Shigella, Pseudomonas, Methylobacterium, Molicutes, Proteus*	Logistic regression using age, sex and metagenomic biomarkers selected by statistical analysis	AUC: 0.95, Sen: 0.90, Spe: 1.00, Acc: 0.93	Kim et al.^90^
				Logistic regression using age, sex and metagenomic and metabolomic biomarkers selected by statistical analysis	AUC: 1.00, Sen: 1.00, Spe: 1.00, Acc: 1.00	Kim et al.^90^
Atopic dermatitis	Blood	*Escherichia-Shigella, Enterococcus, Alistipes, Klebsiella, Veillonella, Bifidobacterium, Akkermansia, Bacteroides*	*Acinetobacter, Pseudomonas, Parabacteroides, Proteus, Prevotella, Dialister, Rhizobium, Sphingomonas, Staphylococcus*	Logistic regression using biomarkers selected by LEfSe	AUC: 1.00, Sen: 1.00, Spe: 1.00, Acc: 1.00	Yang et al.^89^
	Urine	*Pseudomonas, Alicyclobacillus, Propionibacterium, Corynebacterium*	*Lactobacillus, Leuconostoc, Lactococcus, Bradyrhizobium*	–	–	Kim et al.^107^
	Skin washing fluid	*Staphylococcus, Pseudomonas, Streptococcus, Acinetobacter*	*Alcaligenaceae, Sediminibacterium, Lactococcus, Phaeospirillum, Rhodococcus, Lactobacillus, Methylobacterium*	–	–	Kim et al.^122^
Asthma	Blood	*Klebsiella, Bacteroides, Alistipes, Subdoligranulum, Bifidobacterium, Faecalibacterium, Veilonella, Eubacterium, Parabacteroides, Prevotella*	*Pseudomonas, Akkermansia, Citrobacter, Staphylococcus, Micrococcus, Acinetobacter, Lactobacillus, Corynebacterium, Sphingomonas, Propionibacterium, Cupriavidus, Streptococcus*	Logistic regression using biomarkers selected by LEfSe with age and sex as covariates	AUC: 0.97, Sen: 0.92, Spe: 0.93, Acc: 0.92	Lee et al.^88^
		*–*	*–*	Logistic regression using antibacterial EV IgG, IgG1, and IgG4 with smoking status as a covariate	AUC: 0.78, Sen: 0.65, Spe: 0.88, Acc: 0.71	Yang et al.^96^
Autism	Urine	*Halomonas, Streptococcus, Rhodococcus, Bacteroidales S24-7, Akkermansia*,	*Pseudomonas, Sphingomonas, Agrobacterium, Achromobacter*	–	–	Lee et al.^123^
Bipolar depressive disorder	Blood	*Faecalibacterium, Dialister, Klebsiella, Bacteroidales S24-7, Escherichia-Shigella, Ruminococcus, Alistipes, Prevotella*		–	–	Rhee et al.^124^
Major depressive disorder	Blood	*Dialister, Faecalibacterium, Prevotella, Alistipes, Bacteroidales S24-7, Corynebacteriaceae, Escherichia-Shigella*	*Pseudomonas*	–	–	Rhee et al.^124^
Brain tumor	Blood	*Turicibacter, Lactococcus, Lactobacillus, Staphylococcus, Peptoclostridium, Diaphorobacter, Klebsiella, Propionibacterium, Acinetobacter, Salmonella*	*Stenotrophomonas, Sphingomonas, Actinomyces, Streptococcus, Bifidobacterium, Knoellia, Pseudomonas, Corynebacterium, Veillonella*	Logistic regression using biomarkers selected by LEfSe	AUC: 0.97, Sen: 0.93, Spe: 0.90, Acc: 0.91	Yang et al.^82^
				Machine learning algorithm based on the gradient boosting machine (GBM) model	AUC: 0.99, Sen: 1.00, Spe: 0.94	Yang et al.^82^
	Tissue	*Bacteroides, Erysipelatoclostridium*	*Bactroidales S24-7, Prevotella*	–	–	Yang et al.^82^
Chronic rhinitis	Urine	*Propionibacterium, Methylobacterium, Enhydrobacter*	*Achromobacter, Enterobacteriaceae*	–	–	Samra et al.^125^
Allergic rhinitis	Urine	*Methylobacterium*	*Agrobacterium, Achromobacter, Enterobacteriaceae*	–	–	Samra et al.^125^
Atopic asthma	Urine	*Methylobacterium, Sphingomonadaceae*	*Enterobacteriaceae*	–	–	Samra et al.^125^
Hepatocellular carcinoma	Blood	*Staphylococcus, Acinetobacter*	*Pseudomonas, Streptococcus*	Logistic regression using age, sex and biomarkers selected by statistical analysis	AUC: 0.88, Sen: 0.73, Spe: 0.85, Acc: 0.82	Cho et al.^91^
Biliary tract cancer	Blood	*Ralstonia*	*Corynebacterium, Comamonas*	Logistic regression using stepwise selection with age and sex as covariates	AUC: 1.00, Sen: 1.00, Spe: 1.00, Acc: 1.00	Lee et al.^94^
Preterm birth	Blood	*Bacteroides, Lactobacillus, Sphingomonas, Rhizobium*	*Delftia, Pseudomonas, Stenotrophomonas*	–	–	You et al.^126^
Alcoholic hepatitis	Feces	*Veillonella, Lactobacillales*	*Eubacterium, Oscillibacter, Christensenellaceae*	–	–	Kim et al.^127^
COPD	Blood	*–*	*–*	Logistic regression using antibacterial EV IgG, IgG1, and IgG4 with smoking status as a covariate	AUC: 0.79, Sen: 0.90, Spe: 0.61, Acc: 0.81	Yang et al.^96^
Gastric cancer	Urine	*Corynebacterium 1, Neisseria, Fusobacterium, Diaphorobacter, Actinomyces, Porphyromonas, Cloacibacterium, Peptoniphilus*	*Acinetobacter, Staphylococcus, Bifidobacterium, Sphingomonas*	Logistic regression using metagenomic biomarkers selected by statistical analysis	AUC: 0.82, Sen: 0.68, Spe: 0.85, Acc: 0.76	Park et al.^92^
Lung cancer	Blood	*–*	*–*	Logistic regression using antibacterial EV IgG, IgG1, and IgG4 with smoking status as a covariate	Auc: 0.81, Sen: 0.85, Spe: 0.61, Acc: 0.80	Yang et al.^96^
Lung cancer (from COPD)	Blood	*–*	*–*	Logistic regression using antibacterial EV IgG, IgG1, and IgG4 with smoking status as a covariate	AUC: 0.74, Sen 0.69, Spe: 0.69, Acc: 0.69	Yang et al.^96^
Ovarian cancer (from benign ovarian tumor)	Blood	*Acinetobacter*		Logistic regression using biomarkers, age, serum CA-125 levels, and *Acinetobacter* EVs selected by statistical analysis	AUC: 0.85, Sen: 0.82, Spe: 0.68	Kim et al.^95^
Pancreatic cancer	Blood	*Ruminococcaceae UCG-014, Lachnospiraceae NK4A136 group, Akkermansia, Turicibacter, Ruminiclostridium, Lachnospiraceae UCG-001*	*Stenotrophomonas, Sphingomonas, Propionibacterium, Corynebacterium 1*	Logistic regression using age, sex and biomarkers selected by statistical analysis	AUC: 1.00, Sen: 1.00, Spe: 0.92	Kim et al.^93^

*AUC* area under curve, *Sen* sensitivity, *Spe* specificity, *Acc* accuracy.

The presence of antibodies against MEVs can also provide good information for the diagnosis of disease. The presence of IgG antibodies against EVs in indoor dust is closely associated with asthma, COPD, and LC^61^. The levels of IgGs against pathogenic MEVs, such as EVs from *S. aureus, Acinetobacter baumannii, Enterobacter cloacae*, and *Pseudomonas aeruginosa* abundant in indoor dust, showed good AUC values: 0.78 for asthma, 0.79 for COPD, and 0.81 for LC. Thus, MEV-based diagnostic technologies, including MEV composition assessment and immunoassays to assess MEVs, can provide information on exposure to etiological agents^96^.

### Therapeutic potential of microbial EVs

That commensal bacteria play an essential role in human health is beyond doubt, and it is now widely accepted that humans are holobionts or supra-organisms whose collective metabolic potential exceeds the sum of the individual eukaryotic and prokaryotic components. Probiotics or live biotherapeutics (LBPs) are live microorganisms that, when administered in adequate amounts, confer a health benefit on the host and are widely used in many countries in clinical practice. Paraprobiotics are currently referred to as “modified probiotics”, “inactivated probiotics”, “nonviable probiotics”, or “ghost probiotics”^97–99^. Paraprobiotics are a newly emerging modality, an immobilized version of probiotics that has gained traction in recent years due to concerns about the possibility of low tolerance of probiotics or LBPs, especially in pediatric populations and severely ill or immunocompromised patients^100^. The mechanism of action (MoA) of LBPs and parabiotics is not well understood, although the possible MoAs include immune system regulation and interference with pathogen attachment to host cells. Limited research has found that immobilized parabiotics release key bacterial components, including lipoteichoic acids, peptidoglycans, and exopolysaccharides, that exhibit key immunomodulatory effects and antagonizing effects against pathogens^100^.

Postbiotics, also known as metabiotics, biogenics, or simply metabolites, are soluble factors secreted by live microbes or released after microbial lysis, and they provide physiological benefits to the host^101^. Postbiotics are a newly emerging modality in therapy. Cell-to-cell communication is tightly regulated, and its disruption contributes to disease development. Intercellular communication can occur either via cell-to-cell contact or via soluble factors^102^. Soluble factors include proteins and small molecules that bind specific receptors on target cells, which then trigger a signaling cascade that will affect the cell phenotype. However, more recently, another process also involved in this type of communication has been highlighted. In addition to producing specific factors, microbial cells can send a package of information that is enclosed by a cell membrane, also called MEVs. Moreover, recent scientific evidence has shown that certain MEVs have protective effects against disease development or progression^16,103–105^. Thus, MEVs belong to the category of postbiotics and are a new modality for next-generation therapy to complement current therapeutics, such as small molecules, proteins, monoclonal antibodies, and cell therapeutics (Table 3).

**Table 3:** Therapeutic mechanisms of microbial EVs.

Species	Indication	Mechanism	Ref.
*Akkermansia muciniphila (A. muciniphila)*	Colitis	In vitro pretreatment with *A. muciniphila* EVs induced secretion of the proinflammatory cytokine IL-6 from colon epithelial cells. Additionally, oral application of *A. muciniphila* EVs protected against IBD characteristics, such as bodyweight loss, decreased colon length, and inflammatory cell infiltration of colon wall.	Kang et al.^104^
	Metabolic disease	*A. muciniphila* EV administration enhanced tight junction function, reduced bodyweight gain, and improved glucose tolerance in diabetic mice. Additionally, *A. muciniphila* EVs decreased the gut permeability of LPS-treated Caco-2 cells. The expression of occludin was increased by *A. muciniphila* EV treatment.	Chelakkot et al.^106^
*Bacteroides flagilis (B. flagilis)*	Colitis	PSA in *B. flagilis* EVs induced immunomodulatory effects and prevented experimental colitis. DCs sensed *B. flagilis* EV-associated PSA through TLR2, resulting in enhancement of regulatory T cells and anti-inflammatory cytokine production. DCs treated with *B. flagilis* EVs containing PSA prevented experimental colitis.	Shen et al.^128^
*Bifidobacterium longum (B. longum)*	Food allergy	*B. longum* EVs bound specifically to mast cells and induced apoptosis without affecting T cell immune responses. Furthermore, injection of *B. longum* EVs carrying extracellular solute-binding protein into mice markedly reduced the occurrence of diarrhea in a mouse food allergy model.	Kim et al.^111^
*Bifidobacterium bifidum (B. bifidum)*	Allergen-specific immunotherapy	DCs stimulated by *B. bifidum* EVs strongly promoted differentiation of functional CD25^high^FOXP3^high^CD127^−/low^ Treg cells and induced the higher levels of IL-10 than of the proinflammatory cytokines IFN-γ, TNF-α and IL-17. *B. bifidum* EVs can be potentially used as an effective adjuvant for immunotherapy.	Lopez et al.^129^
*Bacteroides vulgatus (B. vulgatus)*	Systemic disease	BMDCs stimulated by *B. vulgatus* EVs led to the induction of a tolerant semimature phenotype. Thereby, the MAMPs delivered by *B. vulgatus* EVs were crucial for the interaction and the resulting maturation of immune cells. In addition to molecules binding to host TLR4, a yet unknown ligand of TLR2 is indispensable for the conversion of immature BMDCs into a semimature state. Thus, by crossing the epithelial mucus layer and directly contacting host cells, *B. vulgatus* EVs mediated cross-tolerance via the transport of various Toll-like receptor antigens.	Maerz et al.^130^
*Escherichia coli (E. coli)*	Cancer	Administered *E. coli* EVs specifically targeted and accumulated in the tumor tissue and subsequently induced the production of the antitumor cytokines CXCL10 and IFN-γ.	Kim et al.^131^
*Klebsiella pneumonia (K. pneumoniae)*	Breast cancer	*K. pneumoniae* EVs enhanced the antihormonal effects of tamoxifen in MCF7 cells via Cyclin E2 and *p*-ERK.	An et al.^132^
*Lactobacillus plantarum (L. plantarum)*	Atopic dermatitis	In vitro, IL-6 secretion from keratinocytes and macrophages was decreased and cell viability was restored with *L. plantarum* EV treatment. Additionally, in mouse AD models, *L. plantarum* EV administration reduced epidermal thickening and the IL-4 level.	Kim et al.^107^
	Skin inflammation	*L. plantarum* EVs promoted differentiation of human monocytic THP1 cells toward an anti-inflammatory M2 phenotype, specifically, the M2b phenotype, by inducing biased expression of cell surface markers and cytokines associated with M2 macrophages. Pretreatment or posttreatment with *L. plantarum* EVs under inflammatory M1 macrophage-favoring conditions, induced by LPS and IFN-γ, inhibited M1-associated surface marker (*HLA-DRα*) expression. Moreover, *L. plantarum* EVs treatment significantly induced IL-1β, GM-CSF and IL-10 expression in human skin organ cultures. Hence, *L. plantarum* EVs can trigger M2 macrophage polarization in vitro, and induce an anti-inflammatory phenomenon in human skin.	Kim et al.^108^
	Depression	*L. plantarum* EVs could have a role in regulating neuronal function and stress-induced depressive-like behaviors. HT22 cells treated with the stress hormone glucocorticoid had reduced expression of BDNF and Sirt1, whereas *L. plantarum* EVs treatment reversed glucocorticoid-induced decreases in the expression of BDNF and Sirt1. siRNA-mediated knockdown of Sirt1 in HT22 cells decreased BDNF4, a splicing variant of BDNF, and Creb expression, suggesting that Sirt1 plays a role in the *L. plantarum* EV-induced increase in BDNF and Creb expression. *L. plantarum* EV treatment in mice rescued the reduced expression of BDNF, and blocked stress-induced depressive-like behaviors. Furthermore, *L. plantarum* EV injection inhibited the reduced expression of BDNF and Nt4/5 and stress-induced depressive-like behaviors. These results suggest that *L. plantarum* EVs can change the expression of neurotropic factors in the hippocampus and induce antidepressant-like effects in mice with stress-induced depression.	Choi et al.^117^
*Lactobacillus paracasei (L. paracasei)*	Inflammatory bowel disease	In in vitro experiments, *L. paracasei* EVs reduced the expression of the LPS-induced pro-inflammatory cytokines (IL-1α, IL-1β, IL-2, and TNF-α), LPS-induced inflammation in HT29 cells, and the activation of inflammation-associated proteins (COX-2, iNOS, NF-κB, and NO) but increased the expression of the anti-inflammatory cytokines IL-10 and TGF-β. In in vivo mouse experiments, oral administration of *L. paracasei* EVs also protected against DSS-induced colitis by reducing weight loss, maintaining colon length, and decreasing the disease activity index. In addition, *L. paracasei* EVs induced the expression of endoplasmic reticulum stress-associated proteins, while the inhibition of these proteins blocked the anti-inflammatory effects of *L. paracasei* EVs in LPS-treated HT29 cells, restoring the pro-inflammatory effects of LPS. *L. paracasei* EVs attenuated LPS-induced inflammation in the intestine through endoplasmic reticulum stress activation.	Choi et al.^105^
*Lactobacillus kefir (L. kefir), Lactobacillus kefiranofaciens (L. kefiranofaciens), Lactobacillus kefirgranum (L. kefirgranum)*	Inflammatory bowel disease	Treatment of TNF-α-stimulated Caco-2 cells with each kefir-derived *Lactobacillus* EV type (*L. kefir, L. kefiranofaciens, and L. kefirgranum*) reduced both mRNA expression and the IL-8 level in association with inhibition of TNF-α signaling by reducing the phosphorylation of p65, a subunit of NF-κB. Subsequent administration of kefir-derived *Lactobacillus* EVs into IBD mice alleviated bodyweight loss and rectal bleeding and enhanced stool consistency. Histological examination showed that kefir-derived *Lactobacillus* EVs substantially reduced the infiltration of transmural leukocytes and loss of goblet cells within the colon, and the serum level of myeloperoxidase was significantly lower in the EV-treated group than control group.	Seo et al.^109^
*Lactobacillus sakei (L. sakei)*	Immune system disease	IgA production in Peyer’s patch cells was enhanced by *L. sakei* EVs. In addition, stimulation of BMDCs with *L. sakei* EVs increased gene expression of inducible NO synthase, retinaldehyde dehydrogenase 2, and several inflammatory cytokines via TLR2.	Miyoshi et al.^110^
*Lactobacillus rhamnosus (L. rhamnosus)*	Enteric nervous system disease	Ingested labeled *L. rhamnosus* EVs were detected in murine Peyer’s patch DCs within 18 h. After 3 days, Peyer’s patch and mesenteric lymph node DCs assumed a regulatory phenotype and increased functional regulatory CD4^+^25^+^Foxp3^+^ T cells. *L. rhamnosus* EVs similarly induced phenotypic changes in cocultured DCs via multiple pathways including C-type lectin receptors specific intercellular adhesion molecule-3 grabbing nonintegrin-related 1 and Dectin-1, as well as TLR-2 and TLR-9. *L. rhamnosus* EVs also decreased the amplitude of neuronally dependent MMCs in an ex vivo model of peristalsis. Gut epithelial application of *L. rhamnosus* EVs caused an increase in the number of action potentials recorded in adjacent patch-clamped sensory neurons.	AI-Nedawi et al.^133^

*PSA* polysaccharide A, *DC* dendritic cell, *BMDC* bone marrow-derived dendritic cell, *ERK* extracellular signal-regulated kinase, *MAMP* microbe-associated molecular pattern.

#### Therapy for immune and inflammatory diseases

*Akkermansia muciniphila (A. muciniphila)* is considered a beneficial anaerobic bacterium living in the large bowel. A previous study^104^ showed that *A. muciniphila* EVs (AmEVs) were decreased in dextran-induced colitis mice vs. healthy mice. In vitro pretreatment with AmEVs improved the production of the proinflammatory cytokine IL-6 by EcEV-stimulated intestinal epithelial cells. In addition, AmEVs protected against dextran-induced IBD phenotypes, including bodyweight loss, decreased colon length, and inflammatory cell infiltration of the colon wall. Oral therapy with AmEVs protected against LPS-induced gut barrier disruption, and this beneficial effect was mediated by the AMPK signaling pathway^106^.

*Lactobacillus* EVs have the possibility to be developed as a medicine for chronic inflammatory diseases such as IBD and AD^105,107^. *Lactobacillus plantarum (L. plantarum)* EVs (LpEVs) have anti-inflammatory effects and prevent SaEV-induced cell death^107^. Oral administration of LpEVs has a therapeutic effect against SaEV-induced atopic dermatitis^107^. LpEVs promoted the differentiation of human monocytic THP1 cells toward an anti-inflammatory M2 phenotype^108^. Treatment of LpEVs under inflammatory M1 macrophage-favoring conditions with LPS and IFN-γ inhibited M1 macrophage-associated *HLA-DRα* expression^108^. Furthermore, LpEV treatment significantly induced IL-1β, GM-CSF, and IL-10 expression in human skin organ cultures^108^. In the case of IBD, the administration of *Lactobacillus paracasei* EVs reduced weight loss, maintained colon length, decreased the disease activity index, and protected against dextran-induced IBD^105^. *Lactobacillus paracasei* EVs inhibited signaling via NF-kB, which is a major transcription factor inducing expression of the inflammatory cytokines IL-6 and TNF-α, and the expression of inducible nitric oxide synthase and cyclooxygenase-2, which is mediated by the unfolded protein response of the endoplasmic reticulum (ER) (UPR^ER^) against ER stress^105^. Moreover, EVs derived from *Lactobacillus kefir*, *Lactobacillus kefiranofaciens*, and *Lactobacillus kefirgranum* inhibited the production of IL-8 from TNF-α-activated Caco-2 cells, reduced rectal bleeding, and improved stool consistency in IBD mice^109^. In addition, it was reported that *Lactobacillus sakei* EVs play crucial roles in the immune system through enhanced IgA production in Peyer’s patch cells^110^.

*Bifidobacterium longum* EVs were reported to alleviate food allergy symptoms mainly by inducing apoptosis of mast cells in the small intestine^111^. In terms of the components in *Bifidobacterium longum* EVs participating in mast cell apoptosis, extracellular solute-binding protein in the EVs specifically reduced the number of mast cells and alleviated diarrhea in mice with food allergies.

#### Therapy for metabolic diseases

*A. muciniphila* LBP and parabiotics (or lysates) have been shown to be beneficial in reducing fat mass and glycemia in T2D patients. The benefits involved the interaction of the *A. muciniphila* OM protein Amuc_1100 with host cell TLR2^112^. Interestingly, it was shown that AmEVs were decreased in T2D patients vs. HCs^113^. Moreover, oral therapy with AmEVs had a therapeutic effect against metabolic changes, such as the obesity and glucose intolerance induced by a high-fat diet. These metabolic benefits are likely linked to the beneficial effect of AmEVs on gut barrier integrity, which is mainly mediated by AMPK signlaing^113^. Taken together, the beneficial effects of *A. muciniphila* on metabolic phenotypes might be mediated by AmEVs.

#### Immunomodulatory therapy for malignant diseases

Cancer immunotherapies such as immune checkpoint inhibitors and chimeric antigen receptor therapies are now being recognized as a promising approach to overcome various malignant diseases^114^. MEVs are nanosized particles that can easily penetrate the gut mucosal barrier and readily interact with immune cells in the gut, giving them great therapeutic potential in the immunology−oncology area. A previous study reported that systemic administration of genetically modified EcEVs significantly and effectively induced antitumor immune responses and fully eradicated established tumors without notable adverse effects^115^. Furthermore, that study showed that systematically administered EcEVs targeted and accumulated in the tumor tissue and subsequently induced the production of the antitumor cytokines CXCL10 and IFN-γ. Moreover, the study found that IFN-γ-deficient mice did not induce such an EV-mediated immune response, suggesting that this antitumor effect is dependent on IFN-γ.

#### Therapy for CNS diseases

Recent evidence indicates that the gut microbiota plays a key role in regulating mental disorders, but the mechanisms by which the gut microbiota regulates brain function are controversial. *L. plantarum* is a gram-positive bacterium present in dairy, fermented vegetables, and the gut^116^. Recent studies have reported the beneficial effects of *L. plantarum* on stress-induced behavioral dysfunction^117^; for example, it improves cognitive deficits^118^, attenuates anxiety behavior^119^, and reduces oxidative stress markers^120^. In addition, we investigated whether LpEVs have a therapeutic effect against stress-induced depression^117^. The study showed that LpEVs protected against and reversed the depressive behaviors induced by psychological stress. The beneficial effect of LpEVs was linked to the overexpression of neurotrophic factor BDNF and the *Nt4/5* and *Sirt1* genes, which are decreased by the stress hormone glucocorticoid^117^. Collectively, these findings suggest that secreted *L. plantarum* EVs show highly beneficial effects and LpEVs can be used to develop novel therapies that penetrate the BBB and modulate CNS function.

## Concluding remarks

We reviewed MEVs as a new tool for precision medicine, especially focusing on the role of MEVs in disease pathogenesis and as biomarkers and MEV therapeutic potential. The pathogenesis of currently intractable diseases is related to cellular aging and increased reactive oxygen species (ROS). Intracellular ROS cause loss of proteostasis, mitochondrial dysfunction, genome instability, and telomerase exhaustion, thereby leading to aging-related diseases, such as immune, metabolic, and neurodegenerative diseases and cancers. According to changes in disease patterns, medical needs have shifted from caring for sick patients to promoting health, from invasive to noninvasive diagnosis methods, and from toxic or expensive drugs to safe drugs with reasonable costs. To meet medical needs, advances in biological data and AI technologies that enable disease prediction and tailored therapy are needed in the near future.

Despite intense microbiome research, there is still much to learn about the intrinsic mechanisms of microbes, especially those of effector microbial products exchanged between the microbiota and host. An exact understanding of microbiota-to-host communication is essential for developing translation strategies employing microbial products for human health. In this context, the study of MEVs is receiving increasing interest. Evidence from nonclinical studies provides us with an opportunity to develop novel first-in-class therapeutic modalities: microbiome-related nanotherapeutics.

MEVs are commensal nonliving materials that act on the human immune system to induce protective responses. Studies have shown that MEVs are absorbed into and distributed in various organs after oral administration, unlike exosomes (human cell-derived EVs). Furthermore, MEVs have a unique ability to target distant organs and effectively penetrate cellular organelles compared with LBPs (live microbes), which are generally restricted to the mucosal or skin surface. Moreover, MEVs can be conveniently administered orally or topically, as opposed to biologics requiring intravenous infusion or subcutaneous injection. LBPs, exosomes, and MEVs are considered to be promising next-generation therapies to complement or replace current biologics, such as protein, monoclonal antibody, cell, and gene therapies. Among the candidates for next-generation therapies, MEVs offer a variety of advantages over LBPs and exosomes in terms of pharmacology (pharmacokinetics and pharmacodynamics), safety, and manufacturing, as summarized in Table 4.

**Table 4:** Comparison of microbial EV therapy vs. live biotherapeutics (LBP) and human cell EV (exosome) therapies.

	Microbial EVs	Live biotherapeutics (LBP)	Human cell EVs (Exosomes)
Pharmacology (PK/PD)	• Multimodal mechanism of action (MoA) • Nanosized EVs enter systemic circulation • Independent of viability • Targeting of specific organs & cellular organelles • Targeting of distant organs (esp. brain) • Efficacious oral application possible	• No penetration of host cells • Generally restricted to the GI tract • Dependent on viability • Targeting of specific organs unfeasible	• Targeting of distant organs unfeasible • Oral administration unfeasible
Safety	• No proliferation • Minimal safety concerns (commensal derived) • Low potential for epigenetic modification	• Uncontrollable proliferation • Concerns for immune-compromised individuals	• Potential for epigenetic modification
CMC	• Minimal storage & stability issues • High yield • Low cost	• Storage & stability hurdles	• Low yield • High cost

*PK* pharmacokinetic, *PD* pharmacodynamic, *CMC* chemistry, manufacturing, and control.

The study of MEVs is rapidly evolving. In the near future, it is expected that research efforts in this area will contribute to the understanding of the role of MEVs in disease pathogenesis, which will provide an essential, solid basis for precision medicine solutions, such as in vitro diagnostics based on circulating MEV profiling and therapeutics based on EVs from beneficial bacteria as postbiotics.
